# Grip Strength across the Life Course: Normative Data from Twelve British Studies

**DOI:** 10.1371/journal.pone.0113637

**Published:** 2014-12-04

**Authors:** Richard M. Dodds, Holly E. Syddall, Rachel Cooper, Michaela Benzeval, Ian J. Deary, Elaine M. Dennison, Geoff Der, Catharine R. Gale, Hazel M. Inskip, Carol Jagger, Thomas B. Kirkwood, Debbie A. Lawlor, Sian M. Robinson, John M. Starr, Andrew Steptoe, Kate Tilling, Diana Kuh, Cyrus Cooper, Avan Aihie Sayer

**Affiliations:** 1 MRC Lifecourse Epidemiology Unit, University of Southampton, Southampton, United Kingdom; 2 MRC Unit for Lifelong Health and Ageing at UCL, London, United Kingdom; 3 Institute for Social and Economic Research, University of Essex, Colchester, United Kingdom; 4 Centre for Cognitive Ageing and Cognitive Epidemiology, Department of Psychology, University of Edinburgh, Edinburgh, United Kingdom; 5 Social & Public Health Sciences Unit, Medical Research Council, Glasgow, United Kingdom; 6 Institute for Ageing and Health, Newcastle University, Campus for Ageing and Vitality, Newcastle upon Tyne NE4 5PL, United Kingdom; 7 MRC Integrative Epidemiology Unit, University of Bristol, Bristol, United Kingdom; 8 Department of Epidemiology and Public Health, University College London, London, United Kingdom; University of Valencia, Spain

## Abstract

**Introduction:**

Epidemiological studies have shown that weaker grip strength in later life is associated with disability, morbidity, and mortality. Grip strength is a key component of the sarcopenia and frailty phenotypes and yet it is unclear how individual measurements should be interpreted. Our objective was to produce cross-sectional centile values for grip strength across the life course. A secondary objective was to examine the impact of different aspects of measurement protocol.

**Methods:**

We combined 60,803 observations from 49,964 participants (26,687 female) of 12 general population studies in Great Britain. We produced centile curves for ages 4 to 90 and investigated the prevalence of weak grip, defined as strength at least 2.5 SDs below the gender-specific peak mean. We carried out a series of sensitivity analyses to assess the impact of dynamometer type and measurement position (seated or standing).

**Results:**

Our results suggested three overall periods: an increase to peak in early adult life, maintenance through to midlife, and decline from midlife onwards. Males were on average stronger than females from adolescence onwards: males’ peak median grip was 51 kg between ages 29 and 39, compared to 31 kg in females between ages 26 and 42. Weak grip strength, defined as strength at least 2.5 SDs below the gender-specific peak mean, increased sharply with age, reaching a prevalence of 23% in males and 27% in females by age 80. Sensitivity analyses suggested our findings were robust to differences in dynamometer type and measurement position.

**Conclusion:**

This is the first study to provide normative data for grip strength across the life course. These centile values have the potential to inform the clinical assessment of grip strength which is recognised as an important part of the identification of people with sarcopenia and frailty.

## Introduction

Grip strength is associated with a variety of ageing outcomes [1]–[3] and forms a key component of sarcopenia [4] and frailty [5], [6] phenotypes. There is considerable interest in its role as a marker of healthy ageing, as an outcome in intervention studies, and as a potential tool for clinical assessment [7]–[9]. The life course epidemiology framework recognises that factors which promote healthy ageing may operate both by increasing the peak grip strength obtained in early adult life as well as by attenuating decline thereafter [10]. There is therefore a requirement for normative data for grip strength which cover all stages of the life course.

Existing normative data have focussed mainly on older ages [11] with relatively few studies examining childhood, adolescence, and early adult life. Since no studies have measured grip strength at all stages of the life course, it is necessary to combine data from studies at different ages. Bohannon et al [12] have previously combined data from 12 studies in adulthood; however, these studies were predominantly modestly-sized samples drawn from the USA. Cohort and cross-sectional studies of the general population conducted in Great Britain (GB) contain a wealth of grip strength data, which in keeping with clinical practice, have been collected using a variety of measurement protocols.

The objective of this paper was to produce cross-sectional centile values for grip strength across the life course by pooling data from a range of general population studies conducted in GB. A secondary objective was to examine the impact of different aspects of measurement protocol on the centile values obtained.

## Methods

### Data sources

We combined data from 12 studies conducted in GB as shown in Table 1. These were all samples of the general population, with eight studies including individuals from specific regions (SWS [13], ALSPAC [14], T-07 [15], HCS [16], HAS [17], LBC1936 [18], LBC1921 [18] and N85 [19]) and four drawing from one (ELSA [20] and ADNFS [21], [22]) or all three countries of GB (UKHLS [23] and NSHD [24], [25]). All included males and females. When combined, studies’ grip measurements covered ages 4 to 90+ years with measurements occurring between 1990 and 2012. Three studies had prospectively recruited participants at or shortly after birth (SWS, ALSPAC and NSHD) and in SWS, grip strength measurements were also available from the mother during her pregnancy and from her partner. The majority (n = 10) of studies had measured grip strength at one or two waves, with LBC1921 and N85 having data from three and four waves, respectively. All studies had received relevant ethical approval and all participants gave informed consent.

**Table 1:** Study details including protocol used for grip strength.

Study (population) ref(s)	Wave*	N seen†	N with gripmeasure	Birthyear(s)	Year(s) ofdata collection	Age range(years)	Device(s) used/position ref(s)	Repetitions/hands/value used
SWS (children of women incohort study, Southampton)[13]	1	1,035	968	2000−2005	2004−2009	4−5	Jamar/seated [52]	Six/both/max.
	2	522	462	2000−2003	2007−2010	6−7		
ALSPAC (children ofwomen attending antenatalclinics in Bristol and DistrictHealth Authority) [14]	1	7,159	6,701	1991−1992	2003−2005	10−14	Jamar/seated	Six/both/max.
ADNFS (random sample ofEnglish population withsubsample having physicalappraisal) [21], [22]	1	3,024	2,602	1916−1974	1990	16−74	Nottingham electronic/seated [36], [53]	Three (or five if third 10% above best of first two)/dominant in 97.2% (non-dominant ifinjured)/max.
UKHLS (nationallyrepresentative sample of UK‡) [23]	1	15,591	14,678	1908−1996	2010−2012	16−102	Smedley/majority(83.1%) standing [54]	Six/both/max.
SWS (partner’s grip strengthat 19 week visit) [13]	1	1,520	1,265	1941−1985	2002−2005	18−58	Jamar/seated [52]	Six/both/max.
SWS (mother’s grip strengthat 19 weeks pregnant) [13]	1	1,634	1,563	1963−1982	2002−2005	21−40	Jamar/seated [52]	Six/both/max.
T-07 (stratified sample fromCentral Clydeside, GreaterGlasgow, Scotland) [15]	1	923	880	1971−1972	2007−2008	35−37	Jamar/majority(99.0%) standing [55]	Six/both/max.
		991	913	1945−1955		52−62		
		654	587	1929−1933		74−78		
ELSA (participants fromHSE aged 50 or older) [20]	1	7,666¶	7,477	1914§–1952	2004−2005	52−89§	Smedley/majority(80.2%) standing	Six/both/max.
	2	8,210**	7,965	1918||−1970	2008−2009	50−89||	Smedley/majority(81.5%) standing	
NSHD (socially stratifiedsample of all births inEngland, Scotland andWales in one week inMarch 1946) [24], [25]	1	2,984	2,847	1946	1999	53	Nottinghamelectronic/seated [56]	Four/both/max.
	2	2,229	2,069		2006–10	60−64		Six/both/max.
HCS (those born in North,East and West Hertfordshireand still resident when traced) [16]	1	2,997	2,987	1931−1939	1999−2004	59−73	Jamar/seated	Six/both/max.
	2 (East Herts. only)	642	639		2004−2005	65−75		
HAS (as per HCS but NorthHertfordshire only) [17]	1	717	717	1920−1930	1994−1995	63−73	Harpenden/seated	Six/both/max.
	2	294	292		2003−2005	72−83	Jamar/seated	
LBC1936 (participants ofScottish Mental Surveysin 1947 at age 11 andstill resident in Lothianarea of Scotland) [18]	1	1,091	1,086	1936	2004−2007	68−70	Jamar/seated	Six/both/max.
	2	866	865		2007−2010	72−73		
LBC1921 (as perLBC1936 but participantsin 1932 at age 11) [18]	1	550	544	1921	1999−2001	78−80	Jamar/seated [57]	Six/both (values from dominant hand used inanalyses)/max.
	2	321	321		2003−2005	82−84		
	3	237	204		2007−2008	86−87		
N85 (those registeredwith a Newcastle/NorthTyneside general practice) [19]	1	849	819	1921	2006−2007	84−86	Takei digital/standing	Four/both/max.
	2	632	603		2007−2009	85−88		
	3	486	453		2009−2010	87−89		
	4	344	296		2011−2012	89−91		

Studies ordered by age at first wave of data collection, youngest first.

*With measurement of grip strength.

† The number here typically refers to the number of participants seen at the stage of the study where grip strength would normally be measured (e.g. at a clinic visit).

‡ The wave 2 nurse health assessment in which grip strength was measured was only carried out in England, Scotland and Wales.

¶ In the first wave of ELSA to measure grip (wave 2), only core study members (n = 8,780) were eligible to take part in the nurse visit and this was completed in the number shown.

§ 80 individuals were aged 90 or older and their exact age is not available.

**In the second wave of ELSA to measure grip strength (wave 4) only core study members (n = 9,886) core members were eligible to take part in the nurse visit and this was completed in the number shown.

|| 91 individuals were aged 90 or older and their exact age is not available.

ADNFS Allied Dunbar National Fitness Survey, ALSPAC Avon Longitudinal Study of Parents and Children, ELSA English Longitudinal Study of Ageing, HAS Hertfordshire Ageing Study, HCS Hertfordshire Cohort Study, HSE Health Survey for England, LBC1921 and LBC1936 Lothian Birth Cohorts of 1921 and 1936, N85 Newcastle 85+ Study, NSHD Medical Research Council National Survey of Health and Development, SWS Southampton Women’s Survey, T-07 West of Scotland Twenty-07 Study, UKHLS Understanding Society: the UK Household Panel Study.

### Grip strength measurement

Information on the grip strength measurement protocols is shown in Table 1. Seven studies used the Jamar dynamometer (including the second wave of HAS, which used the Harpenden dynamometer at the first wave), two studies (ELSA and UKHLS) used the Smedley dynamometer, two studies used the Nottingham electronic dynamometer (ADNFS and NSHD), and N85 used the Takei dynamometer. The majority (n = 8) of studies measured grip in the seated position for all participants.

All studies took measurements from both hands except ADNFS which used the dominant hand only (except in case of injury), and LBC1921 which measured both hands but provided values from only the dominant hand for analyses. The majority of studies used three trials from each hand, except for N85 and the first wave of NSHD, which used two trials. Taken together, this meant that the total number of grip strength values we could use in analyses varied: either three (ADNFS and LBC1921), four (N85 and the first wave of NSHD) or six (the remainder). We therefore always used the maximum of these values for our analyses, since the maximum is less likely to be affected by the number of trials than the mean [26].

### Statistical analyses

Our main analyses used all available data, including values for individuals who had had grip strength measured at more than one age. We produced gender-specific cross-sectional centiles for grip strength using the Box-Cox Cole and Green (BCCG) distribution (also known as the LMS method [27]) implemented in the Generalised Additive Models for Location, Scale and Shape (GAMLSS) library [28] for the statistical program, R [29]. We used restricted cubic splines to model the relationship between age and each of the three model parameters: the median, variation and skewness. We identified the optimum number of degrees of freedom for each parameter using the GAMLSS command find.hyper. We anticipated a smooth relationship with age and therefore used a maximum number of degrees of freedom of seven and increased the standard penalty. We looked for evidence of kurtosis in the grip strength values by using the Box-Cox power exponential distribution. We modelled the mean and SD of grip at each age using the normal distribution in GAMLSS.

We defined a T-score for grip strength as an individual’s value expressed as a multiple of the number of standard deviations below the peak mean value encountered in young adult life. This is the same as the approach applied to measurements of bone density in the diagnosis of osteoporosis [30], except we used gender-specific peak mean values for grip strength. We explored the gender-specific prevalence of weak grip strength in mid and late adult life in two ways. Firstly, using a T-score for grip strength of equal to or less than −2 as used previously [31], and secondly using a T-score of equal to or less than −2.5, as widely used in the diagnosis of osteoporosis.

We carried out sensitivity analyses by producing further sets of centile curves and comparing these to our main findings. We restricted the data to the first observation for each individual. We produced dynamometer-specific sets of centile curves by allowing the median, variation and skewness curves to vary by dynamometer type. Similarly we considered the impact of the position of grip strength measurement: standing or sitting, with the latter divided into those who were sitting as per protocol and those who chose to sit or were unable to stand. Finally we checked if any one study was unduly influencing the results obtained by excluding each study in turn. To compare each additional model to the main findings, we examined absolute differences for the 10^th^, median and 90^th^ centiles; we considered that a 10 percent difference or less in the centile values at any given age provided evidence of acceptably similar findings. We carried out data management using Stata version 12.0 [32].

## Results

**Figure 1:**
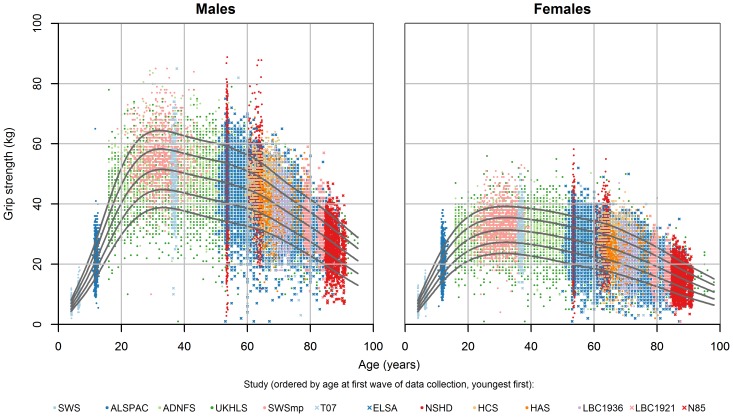
Cross-cohort centile curves for grip strength. Centiles shown 10, 25^th^, 50^th^, 75^th^ and 90^th^. ADNFS Allied Dunbar National Fitness Survey, ALSPAC Avon Longitudinal Study of Parents and Children, ELSA English Longitudinal Study of Ageing, HAS Hertfordshire Ageing Study, HCS Hertfordshire Cohort Study, LBC1921 and LBC1936 Lothian Birth Cohorts of 1921 and 1936, N85 Newcastle 85+ Study, NSHD Medical Research Council National Survey of Health and Development, SWS Southampton Women’s Survey, SWSmp mothers and their partners from the SWS, T-07 West of Scotland Twenty-07 Study, UKHLS Understanding Society: the UK Household Panel Study.

We used a total of 60,803 observations of grip strength from 49,964 participants to produce the centile values for grip strength as shown in Table 2 and Figure 1. Eight of the twelve studies had measured grip strength in mid-late adult life, as reflected by the median age of the observations: 58 years (IQR 36–69 years).

**Table 2:** Normative values for grip strength, stratified by gender.

		Grip strength normative values at age shown (kg)						
Age (years)	Observations *	Centiles					Mean (SD)	
		10th	25th	50th	75th	90th		
**Males**								
** 5**	730	6	7	8	9	10	7.7	(2.9)
** 10**	3222	12	15	17	20	22	17.2	(4.1)
** 15**	288	21	25	29	33	38	29.6	(5.6)
** 20**	354	30	35	40	46	52	41.5	(7.3)
** 25**	574	36	41	48	55	61	48.8	(8.7)
** 30**	984	38	44	51	58	64	51.6	(9.6)
** 35**	1380	39	45	51	58	64	51.6	(10.1)
** 40**	880	38	44	50	57	63	50.3	(10.3)
** 45**	798	36	42	49	56	61	48.8	(10.3)
** 50**	820	35	41	48	54	60	47.6	(10.1)
** 55**	3743	34	40	47	53	59	46.2	(9.8)
** 60**	2683	33	39	45	51	56	44.6	(9.2)
** 65**	3947	31	37	43	48	53	42.3	(8.6)
** 70**	3286	29	34	39	44	49	39.1	(8.1)
** 75**	1883	26	31	35	41	45	35.6	(7.6)
** 80**	1115	23	27	32	37	42	32.2	(7.3)
** 85**	1134	19	24	29	33	38	28.5	(7.0)
** 90**	431	16	20	25	29	33	24.7	(6.8)
** 95+**	5 †							
** (Total)**	(28,257)							
**Females**								
** 5**	700	6	7	8	9	10	8.0	(3.1)
** 10**	3339	12	14	16	19	21	16.7	(3.8)
** 15**	345	17	20	24	27	30	23.9	(4.5)
** 20**	463	21	24	28	32	36	28.4	(5.1)
** 25**	870	23	26	30	35	38	30.6	(5.6)
** 30**	1423	24	27	31	35	39	31.4	(6.0)
** 35**	1785	23	27	31	35	39	31.3	(6.2)
** 40**	968	23	27	31	35	39	30.7	(6.3)
** 45**	952	22	26	30	34	38	29.9	(6.4)
** 50**	1019	21	25	29	33	37	28.7	(6.4)
** 55**	4250	19	23	28	32	35	27.5	(6.4)
** 60**	2943	18	22	27	31	34	26.5	(6.2)
** 65**	4171	17	21	25	29	33	25.3	(6.0)
** 70**	3473	16	20	24	27	31	23.5	(5.7)
** 75**	2135	14	18	21	25	28	21.4	(5.4)
** 80**	1361	13	16	19	23	26	19.1	(5.1)
** 85**	1632	11	14	17	20	23	16.6	(4.7)
** 90**	702	9	11	14	17	20	14.2	(4.4)
** 95+**	15 †							
** (Total)**	(32,546)							

The centiles and mean (SD) values were derived from the GAMLSS models for the exact ages shown.

*Number of grip strength observations refers to the number of individuals at age shown ±2.5 years (to give an indication of the sample size at different ages).

† Limited data were available in the 95+ years category so centile values are not shown.

The centile curves (Figure 1) suggested three overall periods: an increase to peak in early adult life, broad maintenance through to midlife and decline from midlife onwards. Males were stronger on average than females from adolescence onwards; by age 25, males’ median strength was 1.6 times that of females and this ratio increased slightly to 1.7 from age 50 onwards. Males reached a peak median grip of 51 kg (to the nearest whole kg) between ages 29 and 39, compared to the peak female median grip of 31 kg between ages 26 and 42.

The spread of grip strength values relative to the median (the sigma parameter from the BCCG model, an approximation to the coefficient of variation) increased slightly in later life, from 0.20 in the fourth decade in men and women, rising to 0.25 and 0.29 in the ninth decade in men and women, respectively. We found no evidence of skewness or kurtosis in grip strength at any age.

**Figure 2:**
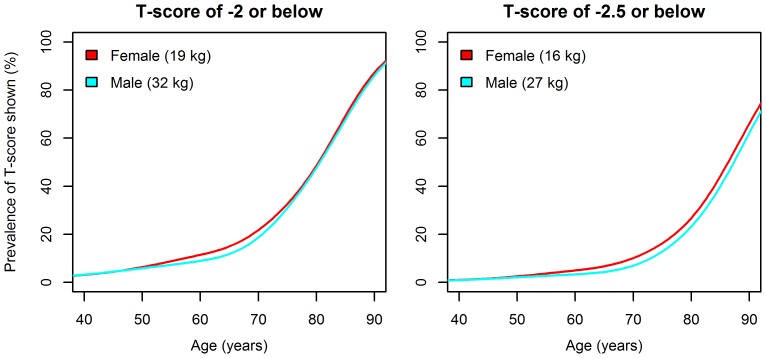
Gender-specific prevalence of weak grip strength based on T-scores of −2 and −2.5. Values shown in brackets are the gender-specific cut-off values calculated by subtracting the relevant number of standard deviations (2 or 2.5) from the young adult peak mean.

Estimated prevalence of weak grip strength in mid and late adult life, defined by gender-specific T-scores of less than or equal to −2 and −2.5, are shown in Figure 2. These were derived relative to the peak mean (SD) for grip strength of 51.9 (9.9) kg in males and 31.4 (6.1) kg in females, both occurring at age 32. Females and males had similar prevalence of weak grip strength during the decline phase. The prevalence of weak grip increased rapidly in late adult life; using a T-score of −2.5, our results suggested that by age 80, around a quarter had weak grip strength (23.0% of males and 26.6% of females).

Sensitivity analyses (see Figures S1, S2, and S3 in File S1) suggested that the centile curves were robust to the inclusion of repeat measurements of grip strength and protocol differences between studies. In comparison to our main results, we generally saw centile differences of less than 10 per cent when restricting the data to the first observation for each individual, and when producing centile curves stratified by dynamometer type. This was also the case for centiles stratified by whether participants were seated (as per protocol) or standing. Those who chose to sit or were unable to stand tended to be weaker and this difference became more pronounced with age until the ninth decade when their 10^th^ centile values approached 10 per cent lower than the combined results. Finally, the centiles produced from analyses excluding each study in turn (not shown) were acceptably similar, except for ALSPAC (males only) and N85 (both males and females); this was perhaps not surprising as the exclusion of each of these studies led to sparse or absent data in the relevant age ranges.

## Discussion

### Main findings

We have combined data from 12 general population studies conducted in GB to produce normative data for grip strength across the life course. We have shown that grip strength increases to a peak in early adult life, and is then followed by a period of broad maintenance prior to decline with increasing age. Our study shows that the strength of males and females is similar until adolescence, after which males began to gain strength more rapidly to a higher peak median of 51 kg between ages 29 and 39, compared to the peak female median grip of 31 kg between ages 26 and 42. Sensitivity analyses demonstrated that the normative data produced by this study are robust to a range of dynamometer types and also to measurement in the seated or standing positions. Our normative data for grip strength across the life course will inform the clinical interpretation of grip strength measurements and will help to establish thresholds for identification of low muscle strength for use in clinical practice and the operationalization of consensus definitions of sarcopenia and frailty.

### Comparison with other studies

Our study is the first to produce normative data for grip strength across the whole life course in GB (or in any other setting, as far as we are aware) so we elected to compare our results with previously published studies of grip strength in international as well as British settings, grouped by the stage(s) of the life course they addressed. We considered differences between previously published mean values and our median values for grip strength at a selection of ages, expressed as a percentage of our value. Normative data from studies identified in childhood and adolescence varied in their relationship to our findings: either broadly similar [33], consistently higher [34] (on average 27%), or similar at young ages and higher at older ages [35] (on average 9% higher overall). However the three previously published studies may not provide reliable estimates of the general population since they contained small numbers of individuals at each given age and gender: at most 43 (mean 22) in each of the ages compared.

We also compared our values to those from four studies addressing adult ages either side of the peak (ages 20–80). Three of these [12], [36], [37] showed agreement with our results, with average differences of around 6%. In one case [36] this is not surprising, since the article reported results from the ADNFS, a study included in our analysis. The second study was the meta-analysis by Bohannon et al [12] which combined data from a range of studies in developed country settings. The third study [37] reported normative data for male participants in the Baltimore Longitudinal Study of Ageing. The normative values from the fourth study [38], based in Switzerland, were on average 11% higher than ours.

Finally, we compared our values to those from three studies which considered age-related differences in grip strength during the decline phase. Normative values from UK Biobank were stratified into eight height groups [39]; in comparison to the average of the middle two groups, our values were on average 7% higher. The TILDA study in Ireland [11] stratified values into two height groups; our values were around 15% higher the average of the groups. Finally a study from Denmark [40] stratified values into five height groups, the middle of which were similar to our own values.

Our results expand on the range of ages as well as the contributing sample sizes of existing studies presenting normative data for grip strength. They also broadly agree with previously published results for adults from developed country settings. Fewer normative data for grip strength in children and adolescents were available for comparison.

We are not aware of any other studies which have compared the centile values obtained from general population samples using different dynamometer types. Several small studies (with 104 or fewer participants) have used comparisons of repeat measurements with two or more dynamometers to investigate whether similar readings are produced. Their findings have varied, with some reporting that readings from different dynamometers are comparable [41]–[44], or can be converted using an equation [45], and others concluding that the limits of agreement are too broad and the devices are not interchangeable in either way [41], [46]. From our results, we conclude that the different dynamometers used produce acceptably similar normative data, albeit within the ages at which measurements were observed.

Similarly, studies investigating the role of measurement position are inconsistent, with one finding no difference [47] and another suggesting that standing produces higher values [48]. Our results show that normative data from studies using the seated and standing positions are comparable, although unsurprisingly individuals who chose not to stand or were unable to do so had weaker grip. Although our centiles appeared to be robust to differences in measurement protocol, this does not detract from the importance of recent calls for standardisation in future data collections [26], [49].

### Clinical relevance of findings

Our findings have confirmed that grip strength increases to a peak in early adult life and is then followed by a period of maintenance prior to decline with increasing age and that this age related decline in grip strength starts as early as the fifth decade of life in both men and women. The life course trajectory identified for grip strength in our study is similar to the well-established life course trajectory of bone mineral density (BMD) [30]. This supports the use of peak values from early adult life to define cut-offs for weak grip at subsequent ages using T-scores. We have used this approach to estimate the prevalence of weak grip based on T-scores of both −2 and −2.5. A T-score of less than or equal to −2 has previously been used by Lauretani et al [31] for grip strength, although the prevalence figures for weak strength that they report using this value, especially those for men, are considerably higher than our own. This difference may have arisen as in their sample, they include 25 men at ages 20–29 with mean (SD) grip 61.1 (10.5) kg. The cut-off for weak grip in men is not stated in their paper but we presume it is then 40 kg (61.1 less 2×10.5) – substantially higher than our own (32kg). By fitting centile curves that span all stages of the life course, we have established more informative peak values on which to base T-scores.

In our data, we still found a high prevalence of weak grip strength based on a T-score of −2 or below (equivalent to 19 kg in females and 32 kg in males, or weaker) with almost half of participants at or below this level at age 80. It may therefore be that a T-score of −2.5 (equivalent to 16 kg in females and 27 kg in males) produces a more discriminatory cut-off for weak grip – with 23.0% of males and 26.6% of females at or below this level at age 80.

It is important that any cut-off values relate to relevant outcomes. Two studies have done this in a cross-sectional fashion. Lauretani et al [31] examined the optimum grip strength values for detecting slow measured walking speed and self-reported difficulty in walking 1 km; they found that grip strength of 30 kg in males and 19 kg in females provided the optimum balance between sensitivity and specificity. Sallinen et al [9] looked at self-reported difficulties with mobility and found similar overall cut-off s: 37 kg in males and 21 kg in females. Clearly there is a need to examine similar relationships in a longitudinal fashion if individual values of grip strength are to be used as a marker of those at risk of adverse outcomes.

### Strengths and limitations

This study had some limitations. First, our data contained a limited range of birth years (at most 32 years) for any given ten year age group. As such the relationships shown with age may partly represent cohort effects [45]. However as the aim of this paper was to produce normative data for current use, the recent period of data collection seems appropriate. Second, our normative data for grip are cross-sectional and are likely to underestimate individual decline; our centiles should therefore not be used for monitoring individual trajectories in grip strength [40], [50], [51]. Third, we have not considered the potential impact of recognised determinants of grip strength, such as height, on the centile values presented. This is an area for future research. Finally selection and loss-to-follow up biases may have influenced our centile values; however we included a wide range of population based studies from different geographical regions of GB and the centile curves were robust to the exclusion of any individual study.

Our study also had many strengths. First, we included data from many large general population studies in GB covering all stages of the life course. Second, we used a modelling approach which allowed grip strength to vary as a smooth function of age and to incorporate any non-normality in grip (skewness or kurtosis). Finally, extensive sensitivity analyses demonstrated that our centile curves for grip strength are robust to differences in the position (seated or standing) and the dynamometer used for measurement.

## Conclusions

In conclusion, we have used existing data from a range of studies conducted in GB to produce centile curves for grip strength across the life course. These centile values have the potential to inform the clinical assessment of grip strength which is recognised as an important part of the identification of people with sarcopenia and frailty.

## Supporting Information

File S1 Figure S1, Centiles from first observation per individual only. Figure S2, Centiles stratified by dynamometer type. Figure S3, Centiles stratified by position of measurement.
(DOCX)

File S2 Data access details for 12 included studies.
(DOCX)
