# Supplementary material for: Grip Strength across the Life Course: Normative Data from Twelve British Studies
Source: PLoS One. 2014 Dec 4;9(12):e113637. doi: 10.1371/journal.pone.0113637 (PMC4256164; doi:10.1371/journal.pone.0113637)
Supplement: File S1 — Figure S1, Centiles from first observation per individual only. Figure S2, Centiles stratified by dynamometer type. Figure S3, Centiles stratified by position of measurement. (DOCX) [file pone.0113637.s001.docx]

**Supplemental material: results of sensitivity analyses**

In the following three sets of graphs, the shaded areas indicate the range 10 percent above and below the values for the 10^th^, 50^th^ (median) and 90^th^ centiles from the main results. The lines then indicate the same centile values from each sensitivity analysis. We considered that centile values from sensitivity analyses within 10 percent of the main results provided evidence of acceptably similar findings. We comment below each graph in instances when this was not the case.

| **Figure S1.** Centiles from first observation per individual only | |
| --- | --- |
| **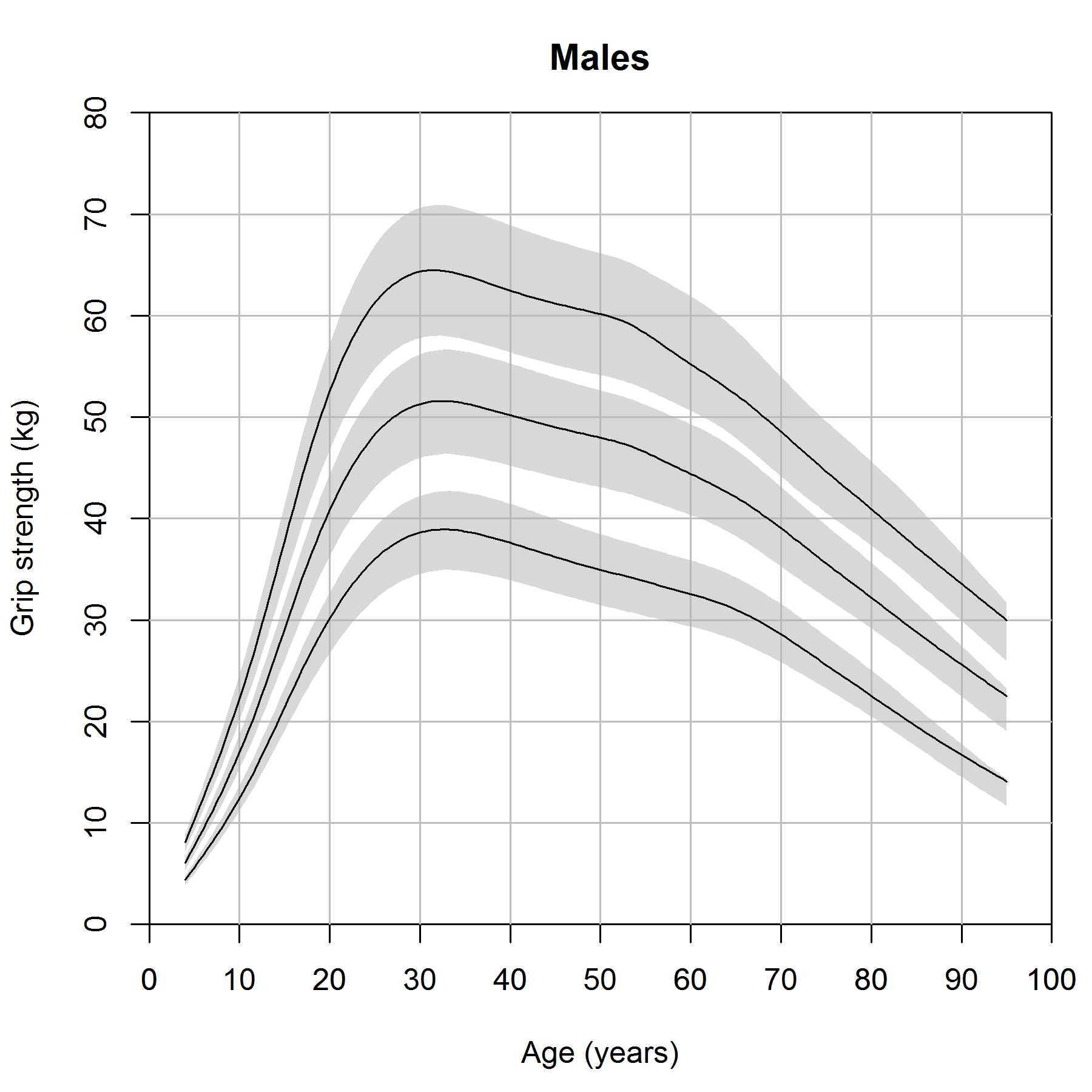** | **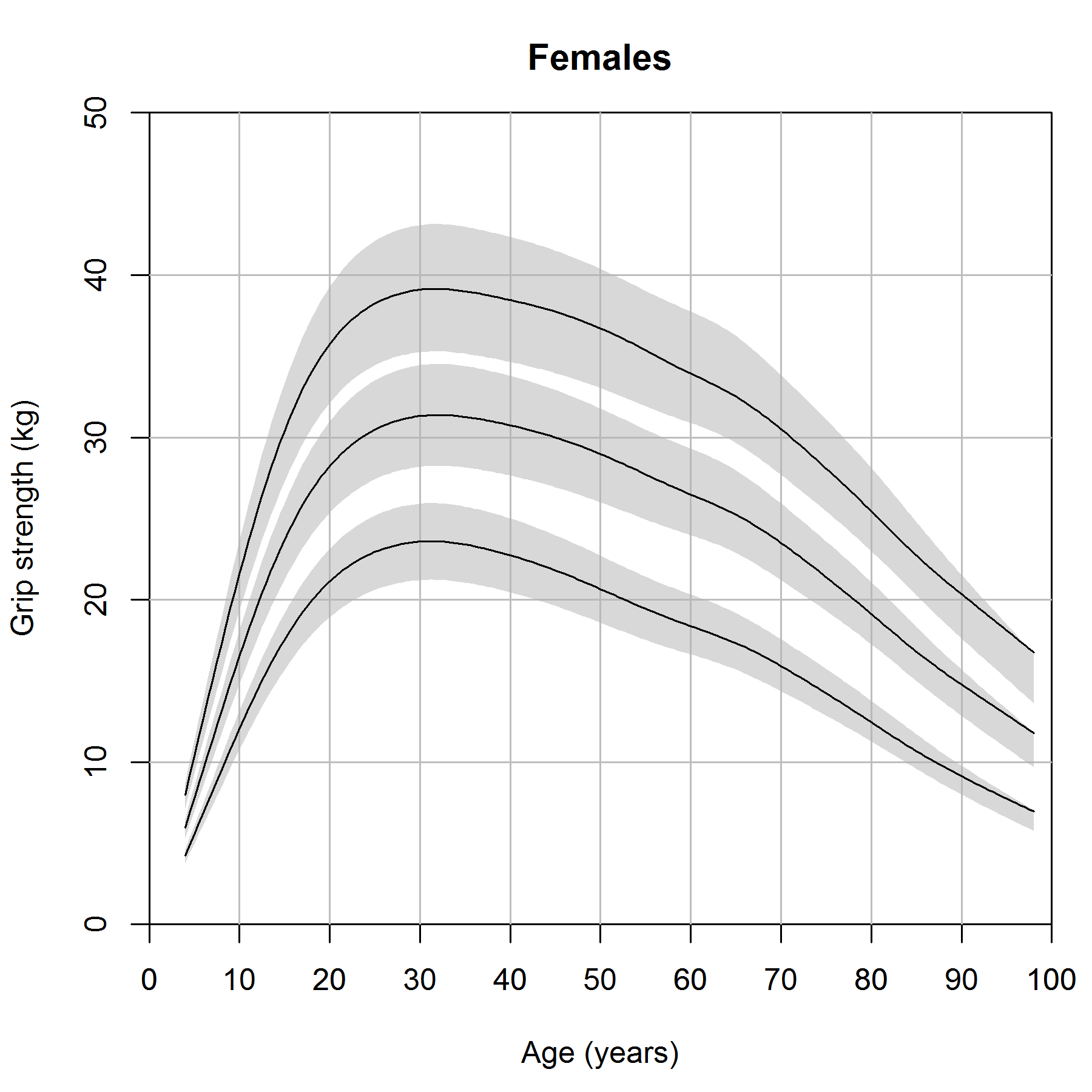** |
| We attributed the slight differences in centile values below age 10 to the lack of data between age c. 4 (the first wave of SWS) and age c. 10 (ALSPAC) which results from the exclusion of repeat grip measurements. | |

| **Figure S2.** Centiles stratified by dynamometer type | |
| --- | --- |
| **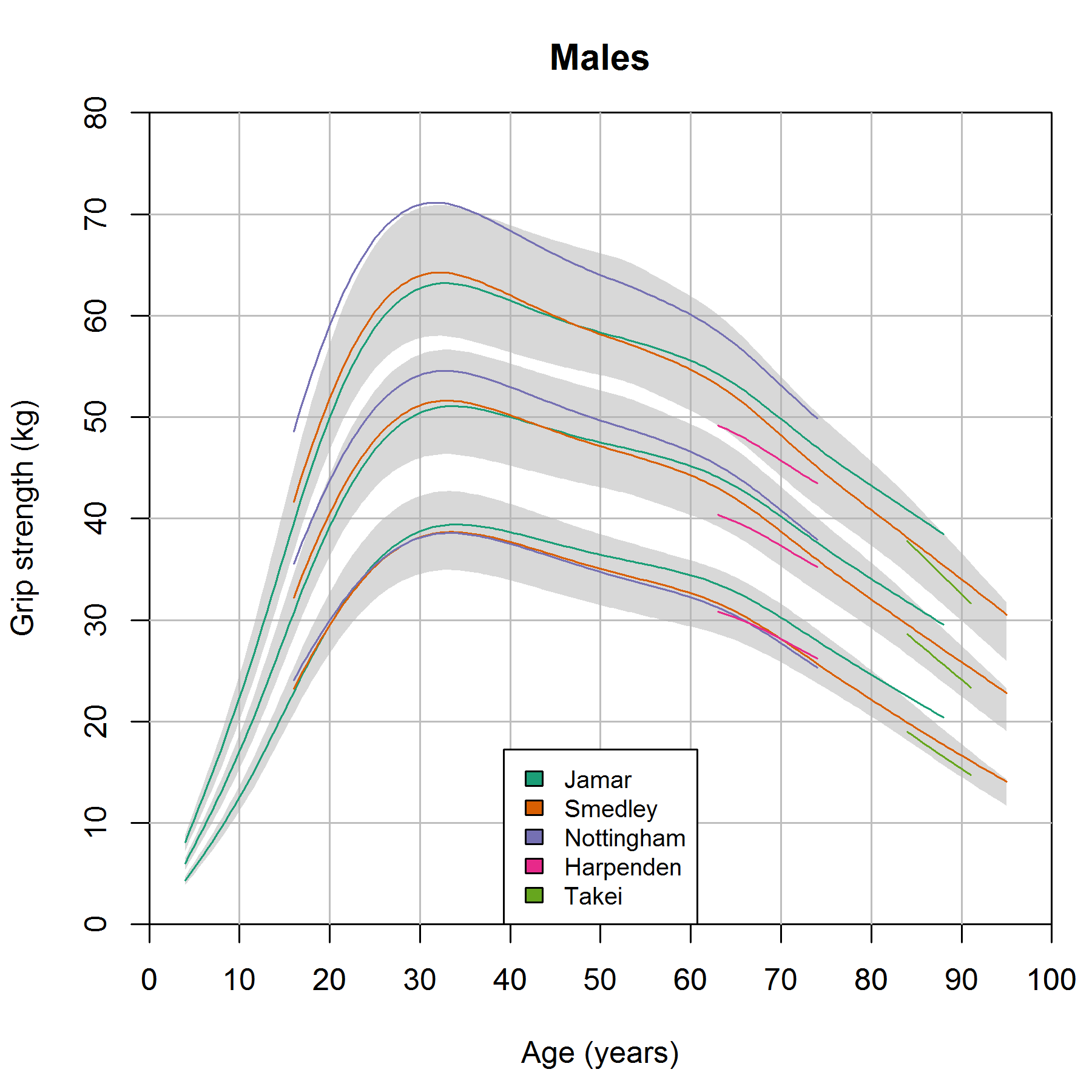** | **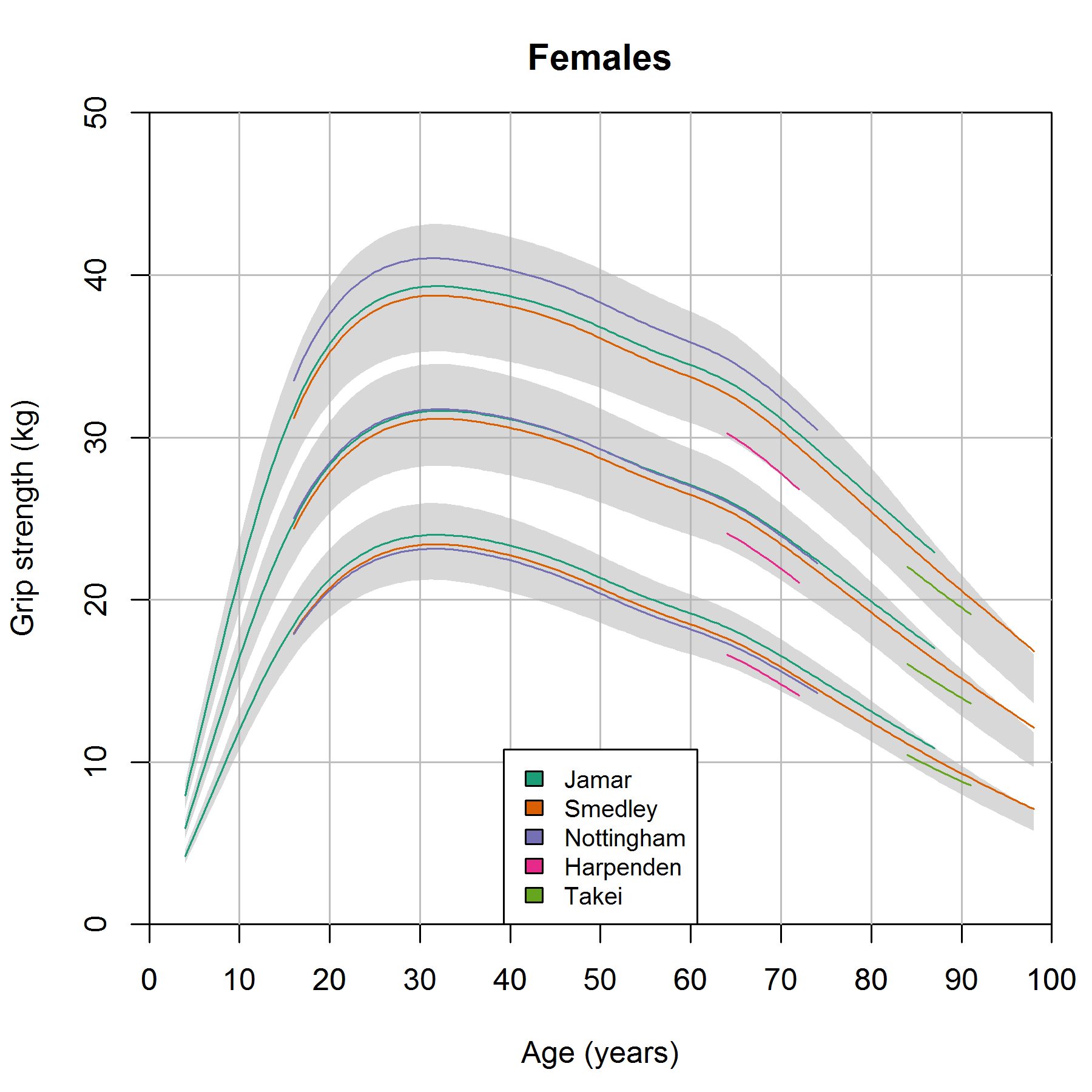** |
| Overall we saw that the dynamometer-specific centiles were similar (to within 10 percent) to the combined results. However the 90^th^ centile values for the Nottingham dynamometer in early adult life in males were slightly elevated. As no other study used the Nottingham dynamometer in this age range, it is unclear if these differences are due to dynamometer or other study factors.  The 10^th^ centile values for the Jamar dynamometer were greater than 10 percent above the combined results between ages 83 and 87 in males; again this period represents observations from a single study (LBC1921) only. | |

| **Figure S3.** Centiles stratified by position of measurement | |
| --- | --- |
| **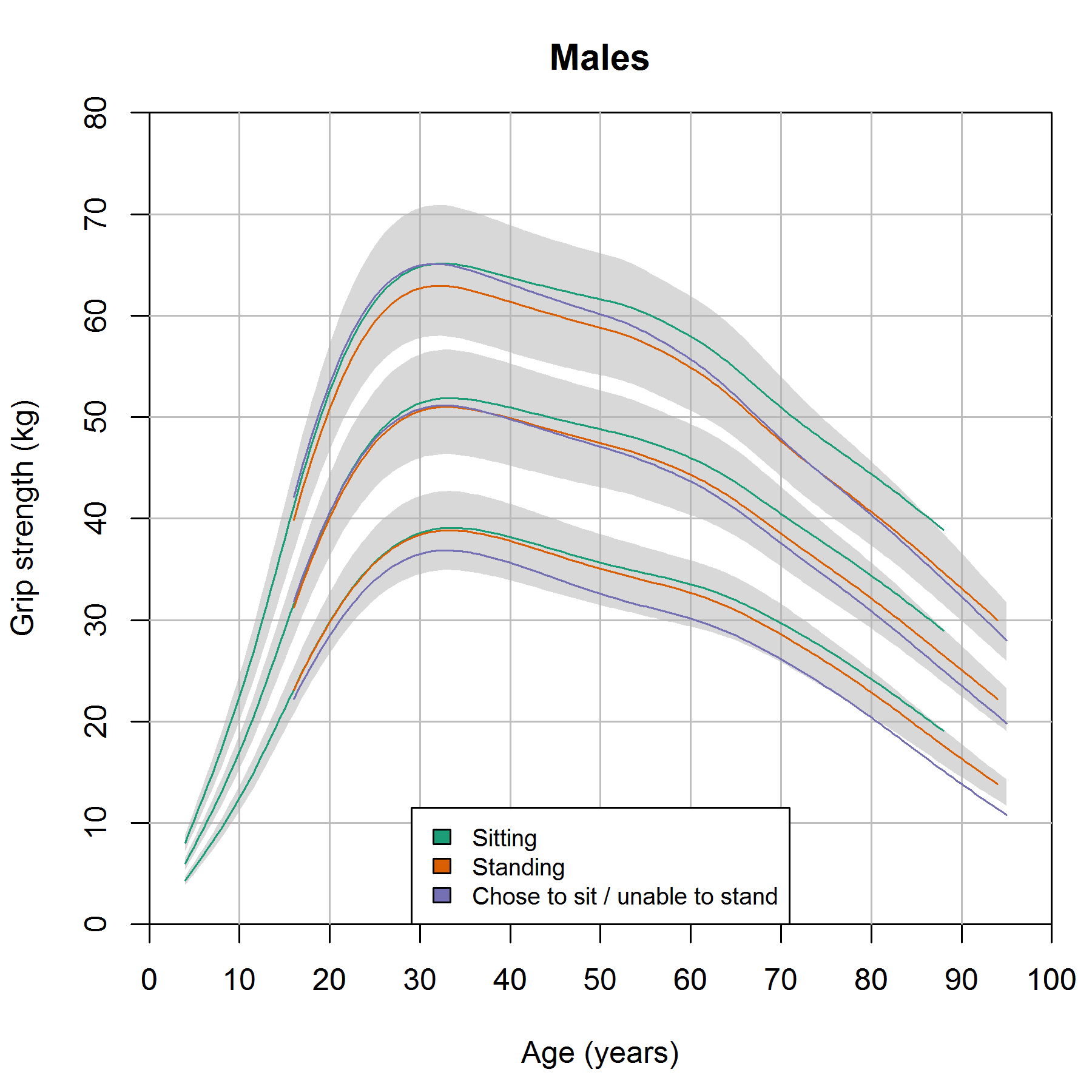** | **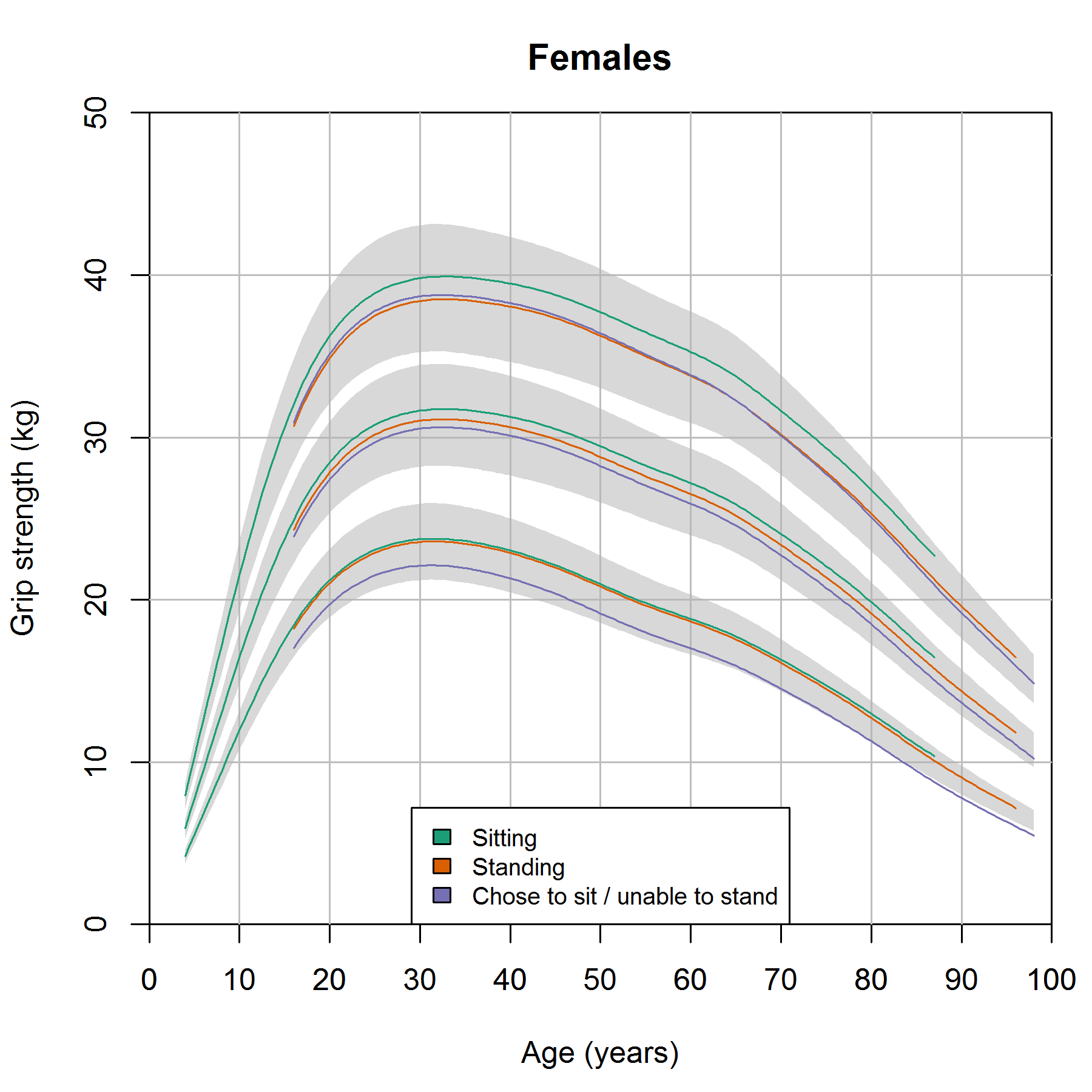** |
| Perhaps unsurprisingly, those who chose to sit or were unable to stand tended to be weaker and this difference became more pronounced with age until the ninth decade when their 10^th^ centile values approached 10 per cent lower than the combined results. | |
