# Supplementary material for: Grip Strength across the Life Course: Normative Data from Twelve British Studies
Source: PLoS One. 2014 Dec 4;9(12):e113637. doi: 10.1371/journal.pone.0113637 (PMC4256164; doi:10.1371/journal.pone.0113637)
Supplement: File S2 — Data access details for 12 included studies. (DOCX) [file pone.0113637.s002.docx]

**Supplemental material: data access arrangements**

The data used in this study are from third-party sources. The table below shows how the data for each study can be accessed, or a request for access can be made as per the study's policy on data-sharing.

| **Study** | **Details of data access / requests** |
| --- | --- |
| Southampton Women’s Survey (SWS) | The initial contact point for collaborations is Professor Cyrus Cooper (cc@mrc.soton.ac.uk). [1] |
| Avon Longitudinal Study of Parents and Children (ALSPAC) | See ALSPAC website <http://www.bristol.ac.uk/alspac/researchers/data-access/> |
| Allied Dunbar National Fitness Survey (ADNFS) | Accessible via the UK Data Service [2] |
| Understanding Society: the UK Household Longitudinal Study (UKHLS) | Accessible via the UK Data Service [3] |
| West of Scotland Twenty-07 Study (T-07) | See the study’s website  <http://2007study.sphsu.mrc.ac.uk/Information-on-data-sharing.html> |
| English Longitudinal Study of Ageing (ELSA) | Accessible via the UK Data Service [4] |
| MRC National Survey of Health and Development (NSHD) | See the study’s website <http://www.nshd.mrc.ac.uk/data.aspx> |
| Hertfordshire Cohort Study (HCS) | Initial enquiries should be made to Cyrus Cooper. (Principal Investigator, cc@mrc.soton.ac.uk) [5] |
| Hertfordshire Ageing Study (HAS) | Initial enquiries should be made to Prof. Avan Aihie Sayer (Principal Investigator, E-mail: aas@mrc.soton.ac.uk). [6] |
| Lothian Birth Cohort of 1936 (LBC1936) | See the study’s website  <http://www.lothianbirthcohort.ed.ac.uk/index.php?option=com_content&view=article&id=15&Itemid=31> |
| Lothian Birth Cohort of 1921 (LBC1921) |  |
| Newcastle 85+ Study (N85) | See the MRC Research Data Gateway <https://www.datagateway.mrc.ac.uk/study/newcastle-85-study>  The Principal Investigator is Professor Tom Kirkwood [tom.kirkwood@ncl.ac.uk](mailto:tom.kirkwood@ncl.ac.uk) |

References (for data access arrangements)

1. Inskip HM, Godfrey KM, Robinson SM, Law CM, Barker DJP, Cooper C. Cohort profile: The Southampton Women’s Survey. Int J Epidemiol. 2006;35(1):42–8.

2. Activity and Health Research Limited. Allied Dunbar National Fitness Survey, 1990 [computer file]. Colchester, Essex: UK Data Archive [distributor], May 1995. SN: 3303, http://dx.doi.org/10.5255/UKDA-SN-3303-1.

3. University of Essex. Institute for Social and Economic Research and National Centre for Social Research, Understanding Society: Waves 1-2, 2009-2011 [computer file]. 4th Edition. Colchester, Essex: UK Data Archive [distributor], December 2012. SN: 6614.

4. Marmot M et al. English Longitudinal Study of Ageing: Wave 0 (1998, 1999 and 2001) and Waves 1-5 (2002-2011) [computer file]. 18th Edition. Colchester, Essex: UK Data Archive [distributor], February 2013. SN: 5050, http://dx.doi.org/10.5255/UKDA-SN-5050-4.

5. Syddall HE, Aihie Sayer A, Dennison EM, Martin HJ, Barker DJP, Cooper C. Cohort Profile: the Hertfordshire Cohort Study. Int. J. Epidemiol. 2005;34(6):1234–42.

6. Syddall HE, Simmonds SJ, Martin HJ, *et al.* Cohort profile: The Hertfordshire Ageing Study (HAS). Int. J. Epidemiol. 2010;39(1):36–43.
